# Role of Bacterial Exopolysaccharides (EPS) in the Fate of the Oil Released during the Deepwater Horizon Oil Spill

**DOI:** 10.1371/journal.pone.0067717

**Published:** 2013-06-27

**Authors:** Tony Gutierrez, David Berry, Tingting Yang, Sara Mishamandani, Luke McKay, Andreas Teske, Michael D. Aitken

**Affiliations:** 1 Department of Environmental Sciences and Engineering, Gillings School of Global Public Health, University of North Carolina, Chapel Hill, North Carolina, United States of America; 2 School of Life Sciences, Heriot-Watt University, Edinburgh, United Kingdom; 3 Department of Microbial Ecology, Vienna Ecology Centre, Faculty of Sciences, University of Vienna, Vienna, Austria; 4 Department of Marine Sciences, University of North Carolina, Chapel Hill, North Carolina, United States of America; University of California, Merced, United States of America

## Abstract

*Halomonas* species are recognized for producing exopolysaccharides (EPS) exhibiting amphiphilic properties that allow these macromolecules to interface with hydrophobic substrates, such as hydrocarbons. There remains a paucity of knowledge, however, on the potential of *Halomonas* EPS to influence the biodegradation of hydrocarbons. In this study, the well-characterized amphiphilic EPS produced by *Halomonas* species strain TG39 was shown to effectively increase the solubilization of aromatic hydrocarbons and enhance their biodegradation by an indigenous microbial community from oil-contaminated surface waters collected during the active phase of the Deepwater Horizon oil spill. Three *Halomonas* strains were isolated from the Deepwater Horizon site, all of which produced EPS with excellent emulsifying qualities and shared high (97-100%) 16S rRNA sequence identity with strain TG39 and other EPS-producing *Halomonas* strains. Analysis of pyrosequence data from surface water samples collected during the spill revealed several distinct *Halomonas* phylotypes, of which some shared a high sequence identity (≥97%) to strain TG39 and the Gulf spill isolates. Other bacterial groups comprising members with well-characterized EPS-producing qualities, such as *Alteromonas* , *Colwellia* and *Pseudoalteromonas* , were also found enriched in surface waters, suggesting that the total pool of EPS in the Gulf during the spill may have been supplemented by these organisms. Roller bottle incubations with one of the *Halomonas* isolates from the Deepwater Horizon spill site demonstrated its ability to effectively produce oil aggregates and emulsify the oil. The enrichment of EPS-producing bacteria during the spill coupled with their capacity to produce amphiphilic EPS is likely to have contributed to the ultimate removal of the oil and to the formation of oil aggregates, which were a dominant feature observed in contaminated surface waters.

## Introduction

Dissolved organic matter (DOM) in the ocean is the largest and possibly least understood pool of carbon - ca. 6.9 x 10^17^ g C, which is comparable in mass to the carbon in atmospheric CO_2_ [1]. Much of this DOM exists as biopolymers (ca. 10-25% of total oceanic DOM) that undergo reversible transition between colloidal and dissolved phases [2,3]. A major fraction of marine DOM derives from the synthesis and release of extracellular polymeric substances or exopolysaccharides (EPS) by bacteria and eukaryotic phytoplankton [4,5]. These are high molecular weight polymers composed mainly of monosaccharides, some of which may contain non-carbohydrate substituents. Several studies have reported large quantities of these bacterial polymers in Antarctic marine environments and around hydrothermal vents, where they are thought to complex with various metal ions and contribute to their mobility and entry into the food web [6 and references therein]. The negative charge associated with carboxyl groups of uronic acids of EPS has been implicated in the capacity of these macromolecules to complex with transition metals [7,8]. EPS from marine bacteria, in particular, are recognized for having much higher levels of uronic acids [9] compared to that found in EPS produced by marine eukaryotic phytoplankton [7] and non-marine bacteria [10]. There is a growing body of evidence to implicate uronic acids of EPS in conferring these macromolecules with an ability to interface with hydrophobic organic chemicals, such as hydrocarbons [11]–[13]. Amino acids and peptides are also often found associated with marine bacterial EPS, which can confer amphiphilic characteristics to these polymers [4,11,14]. The potential significance of marine bacterial EPS to influencing the fate and ultimate degradation of hydrocarbon pollutants in the ocean, particularly during oil spills, remains largely unknown.

The explosion and sinking of the Deepwater Horizon oil rig on April 20, 2010, led to the release of massive quantities of crude oil into the northern Gulf of Mexico, resulting in what has been deemed the worst accidental marine oil spill in the history of the oil and gas industry. The response and complex shifts observed in the microbial communities within and adjacent to waters impacted by the spill have been reported in several publications. Notably, the enrichment of a specific cluster of bacteria within the Order *Oceanospirillales* – organisms associated with aliphatic hydrocarbon degradation – was found initially (late May 2010) dominating an oil plume [15] that had been identified ca. 1000 and 1300 m depth [16]. Dominance of the *Oceanospirillales* in the plume was succeeded by members affiliated to *Colwellia* and *Cycloclasticus* by early June 2010 [17]. This succession from aliphatic hydrocarbon-degrading bacteria (*Oceanospirillales*) to obligate degraders of aromatic hydrocarbons ( *Cycloclasticus* ) and psychrophilic hydrocarbon-degrading generalists ( *Colwellia* ) closely resembles the microbial dynamics observed in seawater following enrichment with crude oil [18]–[23]. Several other bacterial groups comprising members with EPS-producing qualities, notably *Halomonas* , *Alteromonas* and *Pseudoalteromonas* , were also enriched within the water column [15,17]. However, one distinctive feature that has received little attention and remains poorly understood was the observed formation of oil aggregates that were abundant on the surface and within deep water oil plumes [24]. Oil aggregates can be broadly defined as floating gelatinous or “fluffy” material, which, by nature of their “stickiness”, can contain embedded droplets of oil. Oil aggregates representing those formed under conditions simulating the Gulf spill were shown to have a glycoprotein composition – i.e. made up largely of carbohydrate and protein [25]. Bacteria can contribute large quantities of EPS to the total DOM pool in the ocean [26], a large fraction of which can be of glycoprotein composition [3,27]. We therefore hypothesized that EPS-producing bacteria enriched during the spill had contributed to the formation of oil aggregates, as well as to the fate of the oil by influencing the dissolution, bioavailability and ultimate degradation of hydrocarbons by indigenous oil-degrading communities. Whilst oil-aggregate formation at Deepwater Horizon has been shown to be associated with the activities of indigenous microbial communities [25,28], the identity of key species involved in potentially triggering this process remains unknown.

In addition to the enrichment of halomonads in the deep water oil plume at the Gulf spill site [15], these organisms have also been shown to become enriched in laboratory enrichments and in the field after exposure to hydrocarbons [29,32]. *Halomonas* species are slight to moderately halophilic and oligotrophic organisms that are ubiquitous in marine and hypersaline environments [33], many of which are recognized for producing moderate to large quantities of EPS that can exhibit amphiphilic, or biosurfactant-like, properties. Several reports have shown that these polymers effectively emulsify hydrocarbons, crude oils or refined petroleum products [11,31,34]–[39]. In the marine environment, where the fate of spilled oil is both poorly understood and of ecological concern, there is a paucity of knowledge regarding the capacity of EPS produced by these and other EPS-producing bacteria to influence the degradation of hydrocarbons. In particular, polycyclic aromatic hydrocarbons (PAHs) are poorly soluble and generally less amenable to biodegradation compared to their aliphatic counterparts. To circumvent limitations in hydrocarbon bioavailability, some microorganisms produce biosurfactants or bioemulsifiers (e.g. amphiphilic EPS) as a mechanism to increase the bioavailability of these compounds for biodegradation.

In this study, we report on the isolation of three EPS-producing strains of *Halomonas* from oil-contaminated surface waters during the active phase of the Deepwater Horizon oil spill. Two of these isolates, designated strains TGOS-10 and GOS-2, shared 100% 16S rRNA sequence identity to two previously described EPS-producing halomonads – respectively *Halomonas* sp. strain TG39 and TG67 [37]. The chemical composition and associated functional properties of the EPS produced by strain TG39 has been extensively studied [11,37], and therefore served as a model to infer the potential influence of *in situ* bacterial-produced EPS on the fate of the oil released by the Deepwater Horizon blowout. We present new data on the capacity of *Halomonas* EPS to influence the dissolution of aromatic hydrocarbons and enhance the bioavailability of these compounds and rate of biodegradation by the *in situ* sea surface microbial community in the Gulf of Mexico. Using a roller-bottle experimental design, we also provide empirical evidence that implicates indigenous EPS-producing halomonads from Gulf of Mexico waters to having played a role in the formation of oil aggregates during the Deepwater Horizon oil spill.

## Materials and Methods

### Microorganisms and field samples

*Halomonas* sp. strain TG39 was previously isolated from a laboratory culture of an unclassified marine Chrysophyte (CCAP958/1) based on its growth on *n*-hexadecane as a sole carbon and energy source [37]. The strain was routinely grown on a marine broth (ZM/10) composed of ¾-strength naturally aged seawater, peptone (0.05%), yeast extract (0.01%), and supplemented after autoclaving with filter-sterile (0.2 µm) trace elements and vitamins to final concentrations as previously described [40]. A synthetic seawater medium, ONR7a [41], was used to assess the strain’s ability to grow on or mineralize various hydrocarbons.

During a research cruise on the RV *Pelican* on May 5 of 2010, oil-contaminated water samples were collected from the sea surface close to the site of the Deepwater Horizon blow-out. In two follow-up cruises on the RV *Walton Smith* (May 31, 2010) and RV *Cape Hatteras* (Oct. 18, 2010), additional water column samples were collected from the same area. Immediately after collection, the samples were stored at 4°C in their collection vessels (i.e. sealed glass containers) for extraction of DNA, isolation of oil-degraders, and degradation and mineralization experiments. Sample names and sampling locations are shown in Table 1. Sample PE5 was collected 15 days after the blowout from oil-contaminated surface seawater about 0.86 miles from the spill site. Samples B1, B6 and B11 were collected 40 days after the blowout (3.5 mi from the spill site) at 1320 m, 1170 m and 800 m depth which, respectively, represented samples from below, within and above an oil plume that was originally identified between 1000 to 1300 m depth [16]. Sample GIP22 was collected 87 days after capping of the leaky well at 1050 m (37 mi from the spill site) and was used as a reference to represent background levels of the *in situ* microbial community in the Gulf of Mexico.

**Table 1:** Samples collected from the Deepwater Horizon site.

**Sample ID**	**Date** ^a^	**Depth (m)**	**Latitude**	**Longitude**	**Distance (mi)** ^b^
**During spill:**					
PE5 (surface)	May 5	0	28° 44.175 N	88° 22.335 W	0.86
B1 (below plume)	May 31	1320	28° 41.686 N	88° 26.081 W	3.5
B6 (plume)	May 31	1170	28° 41.686 N	88° 26.081 W	3.5
B11 (above plume)	May 31	800	28° 41.686 N	88° 26.081 W	3.5
**Post spill (control):**					
GIP22 (water column)	Oct. 18	1050	28° 40.503 N	87° 39.250 W	37.0

^a^ Collection date in 2010.

^b^ Distance from spill site in miles.

Isolation of PAH-degrading bacteria from the PE5 surface water sample was performed by streaking 5 µl samples onto ONR7a agar plates that were then individually sprayed with phenanthrene, anthracene, pyrene or fluorene [42]. The plates were stored in closed plastic bags in the dark at room temperature. Colonies forming clearing zones (indicating degradation of the hydrocarbon had occurred) were picked, purified and examined for growth on other PAH compounds as per the method of Kiyohara et al. [42]. Purified isolates were sequenced and stored frozen at -80°C in 20% (v/v) glycerol.

### Experiments with *Halomonas* sp. strain TG39 and its produced EPS

#### Growth on hydrocarbons

Because it was identical to one of the isolates from the Gulf spill site (see below) and its cultivation conditions were well-known, strain TG39 was examined for its ability to grow on a variety of hydrocarbon substrates as sole sources of carbon and energy in ONR7a medium. All the compounds used were of reagent quality or better, and were added to final concentrations of between 0.1–0.2% (m/v for solid substrates, v/v for liquid substrates). For organic acids, their sodium salt was used. The compounds tested are listed in Table 2. For phenol and biphenyl, a small crystal was added to ONR7a medium. D-glucose was included as the positive control. For inoculum preparation, strain TG39 was grown in ZM/10 liquid medium to the late exponential phase. The cells were then washed three times with 0.1 M phosphate buffer (pH 7.0), re-suspended using the same buffer (final optical density, 0.3-0.4 at 600 nm) and then inoculated into acid-washed (0.1 N HCl) screw-cap glass tubes (100 x 13 mm) containing 3 ml of ONR7a minimal medium amended with a test carbon source. Growth on benzene, toluene, *p*-xylene, and phenol was also assessed in 250-ml Erlenmeyer flasks using a method for delivering the volatile hydrocarbon via the vapour phase [43]. Test tube cultures and flasks were incubated with shaking (150 rpm) at 28°C. Growth was monitored spectrophotometrically at 600 nm, or by measuring the change in the total protein content over time using the BCA protein assay kit (Sigma, St. Louis, MO) with bovine serum albumin as the standard. Non-inoculated media, and media inoculated with cells but no added carbon source, were included as controls. All experiments were performed in triplicate.

**Table 2:** Growth on and mineralization of some aliphatic and aromatic hydrocarbons by *Halomonas* sp. strain TG39.

**Substrate**	**Growth** ^a^	**Mineralization** ^b^
Phthalate	ND	+
Salicylate	+	+
Catechol	+	ND
Phenol	+c	ND
Benzoate	+	ND
*p*-Cresol	ND	+
o-Cresol	ND	+
Benzene	–	ND
Toluene	–	ND
*p*-Xylene	–	ND
Naphthalene	+	+
Biphenyl	–	ND
Fluorene	–	ND
Phenanthrene	–	–
Anthracene	–	–
Fluoranthene	w	+
Pyrene	w	–
Decane	–	–
Chrysene	w	–
Benzo[a]pyrene	–	–
Hexadecane	+	–

^a^ Determination of growth was assessed spectrophotometrically (OD_600_) or by measuring the total protein concentration in the medium over time, as described in the Materials & Methods. +, positive; –, negative; w, weakly positive; ND, not determined.

b Mineralization was assessed as described in the Materials & Methods section using 14C-labelled compounds.

c Growth occurred only when phenol was supplied via the vapor phase.

#### Mineralization experiments

Experiments were conducted to evaluate the capacity of strain TG39 to completely mineralize ^14^C-labelled hydrocarbon substrates. For this, sterile 40-ml amber-glass EPA vials were prepared, each containing a ^14^C-labeled test compound (to 20,000 dpm) and 2.5 µg of the respective unlabeled test compound in 4.5 ml of ONR7a medium. For the inoculum, cells were grown in 50 ml of ONR7a medium amended with glucose (0.1% w/v). The cells were washed twice with filtered (0.2 µm) natural seawater and then 0.5-ml volumes of the washed cell suspension was inoculated into the vials. For the killed controls, this medium was pre-treated with 85% phosphoric acid to pH <1 prior to inoculation. All treatments were conducted in triplicate. A sterile glass test tube (12 x 75 mm) containing a piece of filter paper saturated with 60 µl of 2 M KOH was inserted into each vial. The vials were sealed with foil-covered Teflon-lined caps and incubated with shaking (100 rpm) at 21°C. The filter paper from each vial was removed every 3 days and the captured ^14^C from any^^14^^ CO_2_ respired was counted on a Packard (Meriden, CT) Tri-Carb liquid scintillation analyzer (model 1900 TR). The KOH-saturated filter paper from each vial was replaced at each sampling point for the duration of the experiment. The percentage of ^14^C mineralized for each compound was calculated by subtracting the triplicate values for the acidified controls from those of the experimental samples and then dividing by the total dpm of ^14^C added.

#### Isolation and purification of strain TG39 EPS

The EPS produced by *Halomonas* sp. TG39 was isolated by growing the strain in ZM/10 supplemented with glucose to a final concentration of 1% (w/v) - i.e. ZM/10+Glc. For this, exponentially-growing cells of strain TG39 were inoculated into several 2-liter Erlenmeyer flasks containing 600 ml of ZM/10+Glc medium. The flasks were incubated with shaking (150 rpm) at 28°C in the dark. After 3 days, the biomass was removed (10,000 x *g*; 30 min) and the supernatant filtered (0.2 µm) to remove residual cells. Isolation of purified EPS was performed as previously described [37]. Briefly, one volume of distilled water was added to the cell-free supernatant to lower its ionic strength as this has been shown to increase the EPS yield [11]. After extensive dialysis, the supernatant was concentrated (Centriplus centrifugal concentrators, 100 kDa molecular-weight cut-off Centricon membranes, Millipore) and the EPS precipitated overnight at 4°C after the addition of KCl (to 7% w/v) and two volumes of cold ethanol. The precipitated EPS was recovered by centrifugation (5000 x *g*; 10 min), extensively dialysed against distilled water and freeze-dried. The resultant EPS was highly purified, the composition of which has been previously characterized and shown to be composed of carbohydrate and protein [11,37].

#### Solubilization assays

The potential of *Halomonas* EPS to increase the solubility of phenanthrene, fluorene, pyrene and biphenyl was evaluated with the EPS from strain TG39 under conditions of high (natural filtered seawater (FSW) at ca. 0.6 M) and low (diluted FSW to ca. 0.1 M) ionic strength. Both solvents were adjusted to pH 8.0 before use. These experiments were adapted from the method of Barkay et al. [44]. Stock solutions of the PAHs, at 2000 mg/l in hexane, were prepared and then distributed into acid-washed (0.1 M HCl) glass test tubes (100 x 13 mm) to yield 60 µg of PAH per tube. The hexane was allowed to evaporate before 3-ml volumes of diluent (0.1 M or 0.6 M FSW) containing increasing concentrations of EPS were added to each tube. The concentration range of polymer tested in the 0.1 M FSW treatments was 0.0 to 1.0 mg/ml. Lower concentrations (0.0 to 0.4 mg/ml) were used in the 0.6 M FSW treatments because the addition of more polymer produced turbid solutions. It was therefore apparent that the polymer’s maximal solubility limit in undiluted FSW was equal to or slightly above 0.4 mg/ml. The tubes were capped and gently vortexed prior to incubating at a slight angle overnight in the dark with shaking (150 rpm; 21°C). All experiments were performed in triplicate. The solutions were then filtered (0.2 µm) to remove non-dissolved PAH crystals. Fractions (2 ml each) of the filtrates were taken into clean glass test tubes containing 2 ml hexane and extracted by vortexing for 2 min. A few drops of a saturated solution of NaCl were added to extractions that formed emulsions. Fractions of the hexane phases were taken into quartz cuvettes and measured spectrophotometrically at 251, 273, 247, and 261 nm for phenanthrene, pyrene, biphenyl, and fluorene, respectively. The *A*_251_, *A*_273_, *A*_247_ and *A*_261_ values were converted to concentrations of phenanthrene, pyrene, biphenyl and fluorene, respectively, using calibration curves that were constructed for each PAH in hexane, as previously described [44].

#### Effect of *Halomonas* EPS on the biodegradation of phenanthrene

Phenanthrene was selected as a model PAH to investigate the influence of the EPS produced by *Halomonas* strain TG39 on the bioavailability and biodegradation of this compound. This was tested using the PE5 water sample collected from the Deepwater Horizon site. For these experiments, sterile 40-ml screw-cap EPA glass vials were prepared, each containing increasing concentrations of EPS -0.0, 0.1, 0.2, 0.4 and 0.8 mg/ml – in 5 ml of ONR7a medium. Each vial was inoculated with 200 µl of cell suspension. In order to maintain a consistent mass transfer of phenanthrene in each vial, a two-phase system employing heptamethylnonane (HMN) was used within which phenanthrene (PHE) was dissolved. HMN is generally considered recalcitrant to biodegradation [45], although some studies have reported its partial breakdown by microorganisms [46,47]. Preliminary experiments in our laboratory with the Gulf water sample yielded no growth on HMN as a sole carbon and energy source. Hence, it was applied in this study as a delivery system for transferring phenanthrene into the aqueous phase. For this, 2 ml of the HMN-PHE solution was carefully dispensed above the aqueous phase in each vial in order to maintain the surface area of the oil–water interface constant and standardized between treatments. All the vials were incubated with gentle rotary shaking (100 rpm) at 21°C for a period of 12 days. Each treatment was performed in triplicate. Sampling was performed by taking 2.5-µl volumes from the HMN-PHE phase in each vial at the time of inoculation and thereafter as indicated. The samples were immediately dissolved in an appropriate volume of ethyl acetate and their absorbance measured spectrophotometrically at 251 nm. The *A*_251_ values were converted to concentrations of phenanthrene using 63,000 liters mol^-1^ cm^-1^ as the extinction coefficient [48]. A decrease in the concentration of phenanthrene was attributed to its degradation in the aqueous phase. The purified EPS did not serve as a source of carbon and energy since control experiments containing only the Gulf water sample and purified EPS did not yield any significant growth (results not shown).

### Experiments with EPS-producing halomonads from Deepwater Horizon

#### Molecular analysis

Total genomic DNA from bacterial isolates was recovered using a Wizard genomic DNA purification kit (Promega, Madison, WI), according to the manufacturer’s instructions. 16S rRNA genes were amplified by PCR with primers 27f [49] and 1492r [50], and then sequenced at the University of North Carolina Genome Analysis Facility. Sequences were analyzed using the program Sequencher 4.8 (Gene Codes Corp., Ann Arbor, MI) and submitted to GenBank. The BLAST search program and RDP-II [51] were used to check for close relatives and phylogenetic affiliation. The search results were used as a guide for tree construction (see below).

#### Emulsification assays

Strains isolated from the PE5 oil-contaminated water sample were incubated in ONR7a medium (120 rpm; 21°C) supplemented with weathered crude oil (2% v/v) that had been collected from contaminated surface water at the Deepwater Horizon site. After 4 days incubation, emulsification assays were performed on cell-free samples of the cultures against fresh aliquots of the oil, as previously described [37]. Briefly, the samples were mixed with an equal volume of *n*-hexadecane in acid-washed (0.1 N HCl) screw-cap glass tubes (100 x 13 mm), manually shaken (15 s) and vortexed (15 s) to homogeneity, left to stand for 10 min, shaken as before, and the height of the emulsion layer – expressed as Emulsification Index, EI_24_ – measured after allowing the mixture to stand for 24 h at 21°C.

#### Roller-bottle incubation experiments

The potential of *Halomonas* sp. strain TGOS-10 in promoting the formation of oil aggregates was investigated using a roller-bottle design similar to that used by Ziervogel et al. [25]. This type of experimental setup maintains the system in a constant gentle turbulence, thereby reducing the potential of particles to settle on the container walls [52] and simulates conditions near the sea surface. *Halomonas* sp. strain TGOS-10 was selected to test this hypothesis because it is an EPS-producer and its 16S rRNA sequence was found to represent ca. 40% of total *Halomonas* fragment reads in the surface slick (PE5) pyrosequence library (see below). Filtered naturally-aged seawater (FSW) was autoclaved and utilized in these experiments in order to directly associate the formation of any aggregates and/or emulsions to this strain.

Three roller-bottle experiments were run in duplicate. The first involved the use of inactive/non-respiring cells (Na-azide treated) to examine the potential of the cell surface alone to promote the formation of oil aggregates or the emulsification of the oil (Experiment I). The second employed using cell-free fractions to determine if any extracellularly-released EPS from these strains promoted the same effect (Experiment II). The third experiment used live cells to evaluate whether cell activity, such as degradation of the oil by the strain, might promote aggregate and/or emulsion formation (Experiment III). The inoculum for each of these experiments was prepared by growing up a large batch of cells in FSW amended with filter-sterilized Gulf of Mexico BP crude oil (0.2 µm; 2% v/v), glucose (to 0.05% w/v final concentration), and a trace elements and vitamin mixture [41]. The cell biomass was recovered by centrifugation (8,000 x g; 20 min) and the supernatant fraction set aside. The cells were washed twice with autoclaved FSW, re-suspended to a final optical density (600 nm) of 0.06, and supplemented with Na-azide (0.01% w/v final concentration) to render the cells inactive for use in Experiment I. Preparation of the cell-free fraction for Experiment II involved removal of all residual cells from the supernatant fraction (collected as described above) by filtration (0.2 µm) and supplementing the filtrate with Na-azide as above. Washed ‘live’ cells (i.e. in the absence of Na-azide treatment) were used (0.06 final OD_600_) for Experiment III. Each of the three experiments was conducted in duplicate using 250-ml Pyrex© glass bottles (38 x 265 mm) which were filled with 200 ml of the respective inoculum fraction, and to each 1 ml of sterile BP crude oil was added to a final oil slick content of 0.5% (v/v). Control incubations were run in parallel using uninoculated autoclaved FSW in the presence or absence of oil. Inoculated controls were also included, but without the addition of oil. The bottles were incubated at 25°C in the dark on a roller table at 3.5 rpm for 12 days. The bottles were periodically placed upright to photographically record the formation of oil aggregates and emulsification of the oil. Samples were also withdrawn for light microscopy or staining with acridine orange [53] for imaging with a FITC filter on a Zeiss Axioscope (Carl Zeiss, Germany).

At the end of these 12-day roller bottle incubations, visible aggregates were carefully withdrawn using glass Pasteur pipettes and transferred to 1.5-ml microtubes for staining with the cationic copper phthalocyanine dye alcian blue (AB) at pH 2.5 [54] or the amino acid-specific dye coomassie brilliant blue G (CBBG) at pH 7.4 [27]. AB is used for staining acidic sugars of EPS, whereas CBBG is used for staining the proteinaceous component of EPS. Following staining, the aggregates were washed by transferring them through several droplets of sterile water prior to their examination under the light microscope.

### Pyrosequencing

Bar-coded 16S rRNA gene pyrosequencing was performed on the water samples from Gulf of Mexico to identify *Halomonas* -related sequences. Ten-fold dilutions of extracted DNA samples in water were used as template for triplicate PCR reactions for each sample. Primer pairs 27f and 338r were modified to incorporate an identical 8-base-pair (bp) bar code sequence unique to each sample and a 2-bp spacer on the 5’ end of the primer sequence [55]. Each 20 µl PCR reaction was run for 25 cycles of 94°C for 45 s, 55°C for 45 s, and 72°C for 1 min on an Eppendorf (Westbury, NY, USA) Mastercycler Gradient thermal cycler before verification of the proper amplicon size on a 1% agarose gel. The triplicate reactions for each sample were pooled and purified with a QIAquick PCR Purification Kit (Qiagen, Valencia, CA, USA) and eluted in 30 µl of 10 mM Tris-Cl (pH 8.5) buffer. The DNA concentration of pooled amplicons was then measured using a NanoDrop ND-3300 Fluorospectrometer (Thermo, Waltham, MA, USA) and Quant-iT Picogreen dsDNA Kit (Invitrogen, Carlsbad, CA, USA) prior to combining into a single sample at a concentration suitable for pyrosequencing. The sample was submitted to the High-Throughput Sequencing Facility at the University of North Carolina-Chapel Hill for sequencing using the 454 Life Sciences Titanium platform (Roche Diagnostics, Branford, CT, USA).

Pyrosequencing reads were trimmed and filtered using the LUCY program with a minimum PHRED score of 27.5 and minimum length of 200 nt to remove low-quality regions and short reads [56]. Reads were de-multiplexed based on the 8 bp bar code sequence, and the primer and bar code regions were removed using QIIME [57]. To form operational taxonomic units the reads were clustered at 97% sequence identity with UCLUST [58] and the most abundant unique read within each cluster was used as its representative sequence. Initial phylogenetic identification was made using BLAST [59] and chimeras were detected with Chimera Slayer [60]. Sequence data were submitted to the European Nucleotide Archive Sequence Read Archive under the study accession number ERP002443.

*Halomonas* sequences were identified by manually aligning the representative sequences in ARB [61] to sequences of cultivated and well-characterized *Halomonas* species. A bootstrapped neighbour-joining phylogenetic tree (with 1000 bootstraps) of near-full-length 16S rRNA sequences was produced using the Jukes-Cantor substitution model and the pyrosequencing reads were then added to this guide tree using the parsimony quick-add function in ARB.

### Statistical analysis

A Student’s *t* test was performed to test for significant differences (*P* < 0.05) in the degradation and solubilization of hydrocarbons between the different treatments.

### Nucleotide sequence accession numbers

The 16S rRNA gene sequence of strains GOS-2, GOS-3a and TGOS-10 were deposited with GenBank under accession numbers JQ246430, JQ246431 and JQ246432, respectively.

## Results

### Hydrocarbon degradation by *Halomonas* sp. strain TG39 and amphiphilic properties of its produced EPS

#### Utilization of hydrocarbons

Table 2 shows the various substrates that were tested for growth or mineralization by the representative strain TG39. Growth was observed on salicylate, catechol, phenol, benzoate, naphthalene and hexadecane. Weak growth was recorded on fluoranthene, pyrene and chrysene, and no growth was measured on decane, benzene, toluene, *p*-xylene, biphenyl, fluorene, phenanthrene, anthracene and benzo[a]pyrene. The liberation of^^14^^ CO_2_ from ^14^C-labeled compounds was also assessed. After 9 days of incubation, significant levels (*P* < 0.05) of salicylate (73.9 ± 1.4%), o-cresol (47.2 ± 14.3%), *p*-cresol (11.8 ± 0.9%), naphthalene (6.1 ± 0.2%), fluoranthene (3.9 ± 0.6%) and phthalate (1.2 ± 0.2%) were mineralized of total added ^14^C-labeled compound when compared to their respective acid-killed controls. Strain TG39 did not significantly mineralize phenanthrene, anthracene, pyrene, benzo[a]pyrene, chrysene, decane and hexadecane compared to their respective acidified controls. The strain’s ability to mineralize *p*-cresol, o-cresol, naphthalene and fluoranthene, and produce weak growth on pyrene, is reflected in its ability to assimilate phthalate, salicylate, catechol and benzoate which are common intermediates in PAH degradation.

#### EPS-mediated solubilization of aromatic hydrocarbons

**Figure 1:**
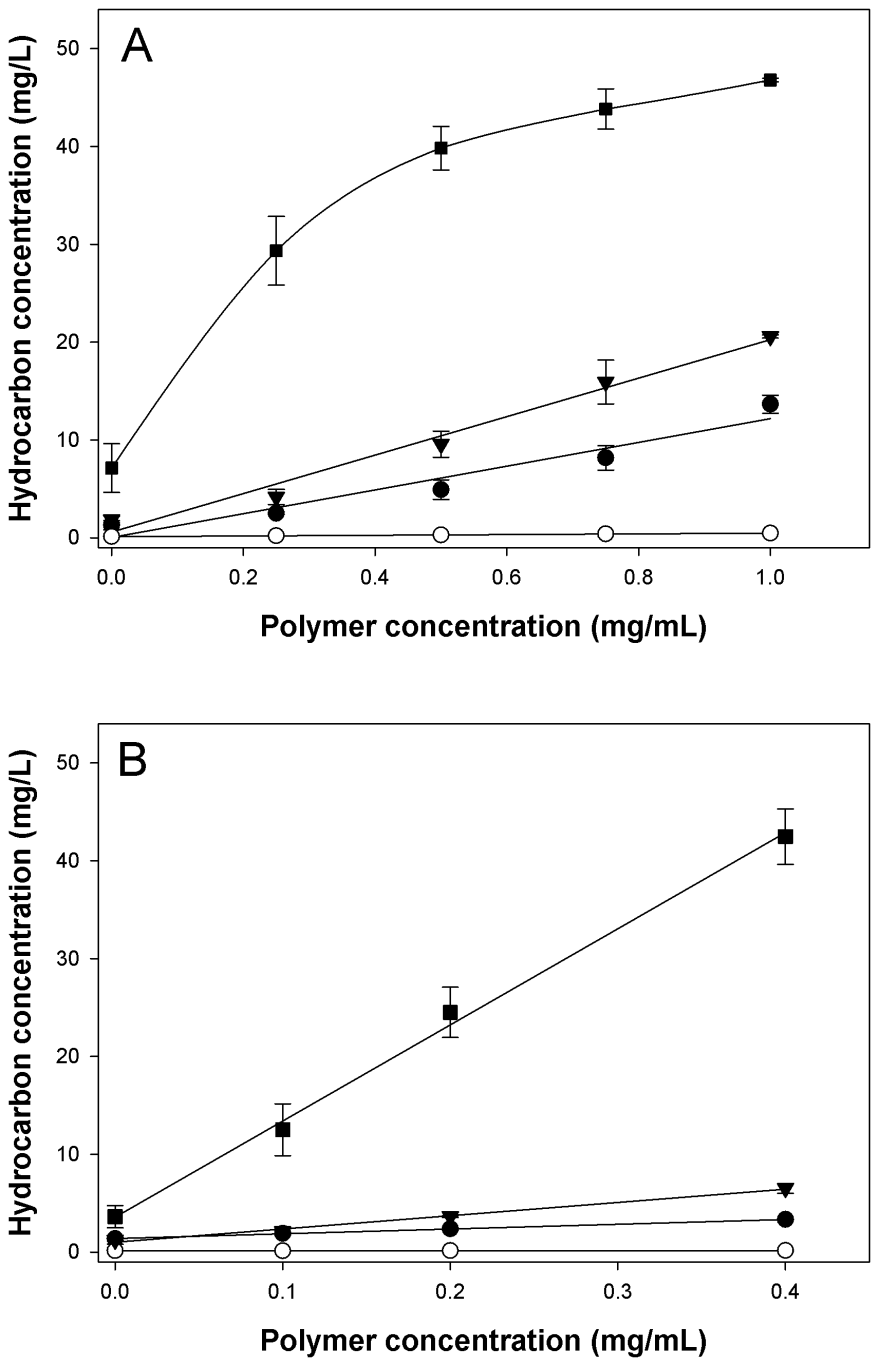
Effect of *Halomonas* strain TG39 exopolysaccharides (EPS) on the dissolution of aromatic hydrocarbons. Relationship of EPS concentration to aromatic hydrocarbon solubilization in diluted filtered seawater (0.1 M; pH 8.0) (A) and in undiluted filtered seawater (0.6 M; pH 8.0) (B). Note difference in scale of x-axis (polymer conc.) between plots. The EPS used was from *Halomonas* sp. strain TG39. Phenanthrene (*solid circles*); pyrene (*open circles*); fluorene (*inverted triangles*); biphenyl (*squares*).

To explore the potential of the *Halomonas* EPS to increase the solubility of aromatic hydrocarbons, solubilization assays were performed with the EPS from strain TG39 using phenanthrene, pyrene, fluorene and biphenyl. These experiments were conducted under conditions of low (0.1 M) and high (0.6 M) ionic strength in order to evaluate the effect of salts on the solubilization of these hydrocarbons as a function of increasing EPS concentration. As shown in Figure 1, the concentrations of the four compounds increased linearly (r^2^ ≥ 0.94) as a function of increasing polymer concentration in both the 0.1 M and 0.6 M FSW treatments, with the exception of biphenyl in the 0.1 M treatment. At a polymer concentration of 0.4 mg/ml, the soluble concentration for each of the four aromatic hydrocarbons was calculated from the linear curves in Figure 1. In the 0.1 M FSW treatment, the soluble concentration of biphenyl was 36.8 mg/L, which is 5-fold higher than in the untreated control (7.2 mg/L); soluble fluorene was measured at 8.5 mg/L, a 4.7-fold increase relative to the control (1.8 mg/L); soluble phenanthrene was measured at 4.9 mg/L, a 3.8-fold increase relative to the control (1.3 mg/L); and soluble pyrene was measured at 0.3 mg/L, a 3-fold increase relative to the control (0.1 mg/L). In the 0.6 M FSW treatment, the soluble concentration of biphenyl was 42.5 mg/L, which is 11.8 times higher than in the untreated control (3.6 mg/L); soluble fluorene was measured at 6.5 mg/L, a 5.4-fold increase relative to the control (1.2 mg/L); soluble phenanthrene was measured at 3.3 mg/L, a 2.4-fold increase relative to the control (1.4 mg/L); and soluble pyrene was measured at 0.2 mg/L, a 2-fold increase relative to the control (0.1 mg/L). Overall, phenanthrene, fluorene and pyrene were more effectively solubilised by the *Halomonas* EPS under conditions of lower ionic strength (i.e. 0.1 M), since the differences between their concentrations at 0.4 mg/ml in the two treatments was found to be significant (*P* < 0.05).

#### Effect of EPS on phenanthrene biodegradation

**Figure 2:**
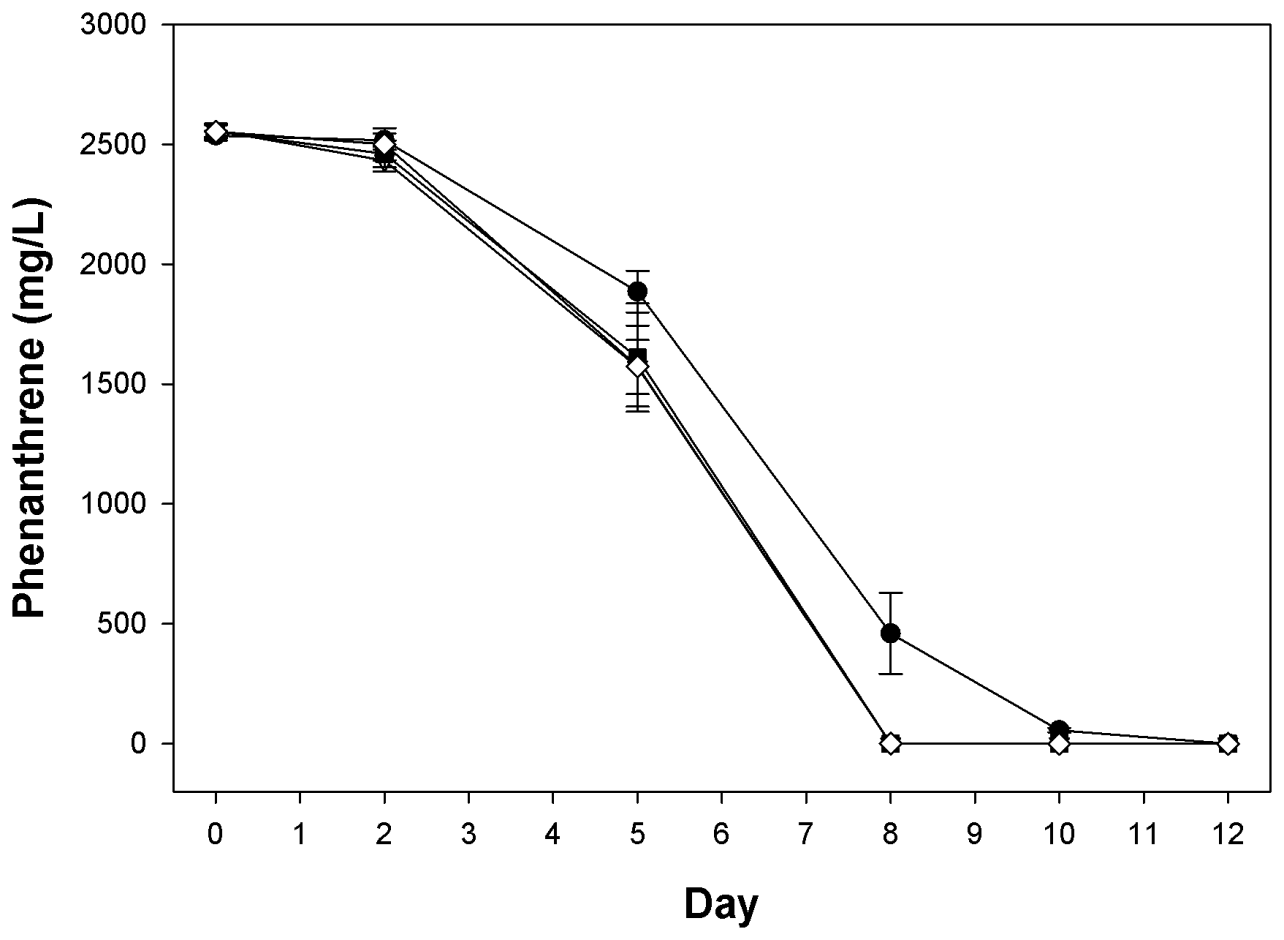
Effect of *Halomonas* strain TG39 exopolysaccharides (EPS) on the degradation of phenanthrene by a Deepwater Horizon microbial community. Residual phenanthrene over time in incubations with a natural microbial community (sample PE5) that had been collected from the Deepwater Horizon site on May 5, 2010. Concentrations of EPS used: 0.0 mg/ml (*circles*); 0.1 mg/ml (*inverted triangles*); 0.2 mg/ml (*squares*); 0.4 mg/ml (*diamonds*).

Figure 2 shows the polymer’s effect on the degradation of phenanthrene by the indigenous microbial community from oil-contaminated surface waters (i.e. sample PE5). Degradation followed first order kinetics in all four treatments with an initial lag of approximately 2 days, followed thereafter by rapid rates of degradation that were higher for the EPS-amended treatments compared to the untreated control. During the time interval between day 2 to day 5, the degradation rates for the three EPS-amended treatments were statistically similar (281–310 mg/l/day; *P* > 0.05), though significantly higher (*P* < 0.05) compared to that of the untreated control (211 mg/l/day) (Supplementary table S1). During the interval between day 5 to day 8, the degradation rate of the untreated control was slightly less (476 mg/l/day) though not significantly different (*P* > 0.05) compared to that of the EPS-amended treatments (526–537 mg/l/day). Complete removal of the phenanthrene in the EPS-amended treatments occurred sooner (i.e. by day 8) than in the untreated control (i.e. 11-12 days). Incubations that were run in parallel showed that the microbial community in the PE5 sample was capable of producing measurable, albeit very weak, growth on the polymer as a carbon source (results not shown).

### EPS-producing bacteria, their abundance at Deepwater Horizon and role in oil-aggregate formation

#### Molecular analysis and characteristics of *Halomonas* *isolates*

Several colonies surrounded by clearing zones grew out on agar plates that had been streaked with the PE5 water sample and sprayed with phenanthrene. Subsequent purification and sequencing identified three distinct *Halomonas* strains – strains GOS-2, GOS-3a and TGOS-10. Colonies forming clearing zones on fluorene-sprayed plates were also observed, but attempts to purify and maintain these organisms proved difficult. No clearing zones were observed on plates sprayed with anthracene or pyrene. Versatility for degrading various PAH compounds was observed with strain TGOS-10 as it was able to grow on different PAHs.

**Figure 3:**
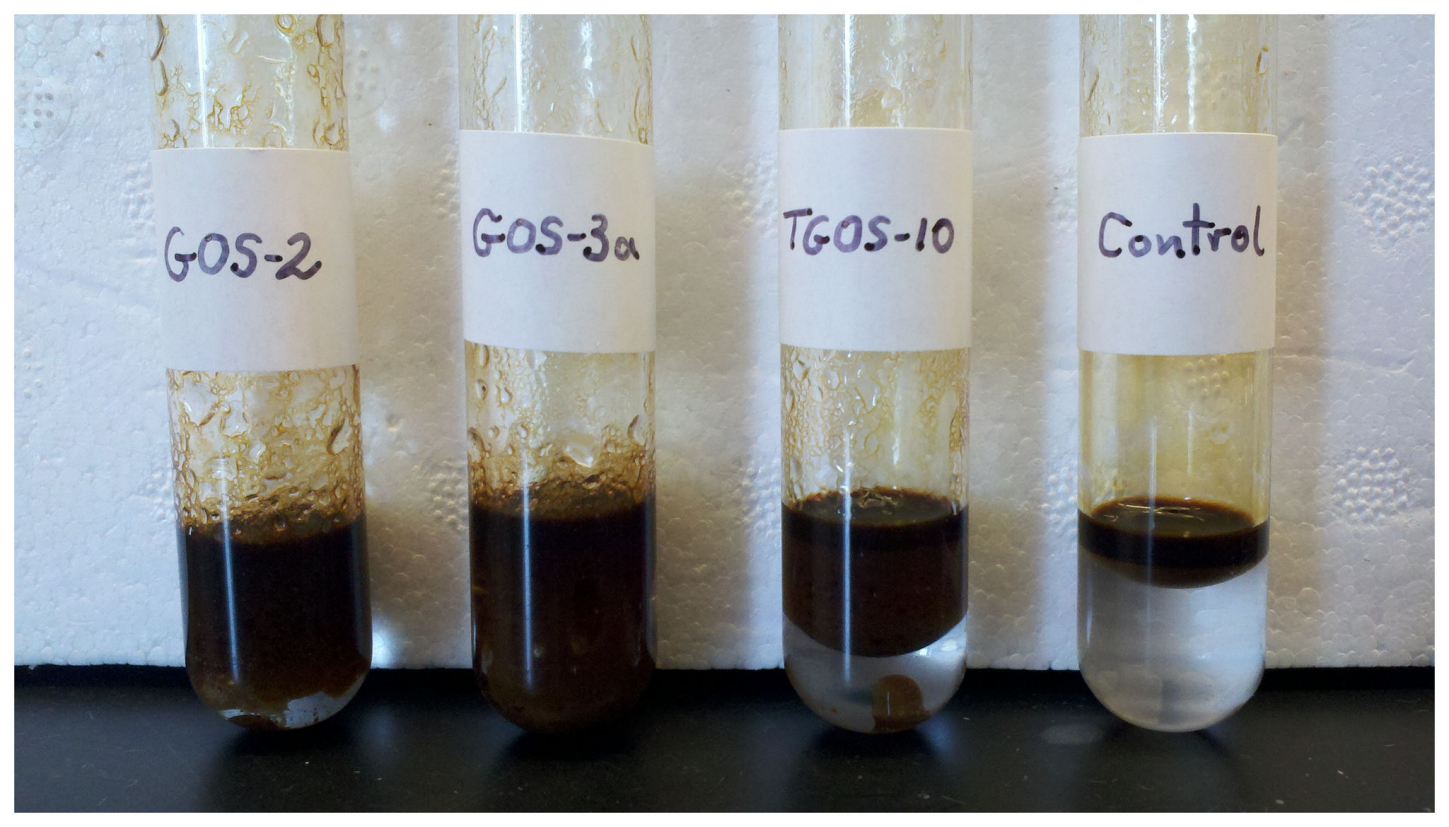
Photos of water-in-oil emulsions formed by cell-free fractions of strains GOS-2, GOS-3a and TGOS-10. Emulsions were conducted of the cell-free fractions after incubation of each of the strains for 4 days in minimal seawater medium (ONR7a) amended with weathered crude oil 2% (w/v) from the Deepwater Horizon spill. Cell-free culture samples were derived after removal of the cells by centrifugation (13,000 x *g*; 10 min) and then filtration (0.2 µm).

During incubation on weathered crude oil (from the Deepwater Horizon spill site), these strains were able to grow on the oil as evidenced by an observed increase in the turbidity of the cultures after 4 days incubation. Cell-free samples from these enrichments were found to produce stable emulsions when tested against the oil. These emulsions appeared as thick viscous emulsions (Figure 3), much like the ‘chocolate mousse’ characteristic of crude oil after being subjected to natural wave and wind action at sea. Conversely, uninoculated medium (i.e. control incubations) did not show an increase in turbidity or emulsification of the oil, and washed cells of these strains did not yield emulsions with the crude oil.

**Figure 4:**
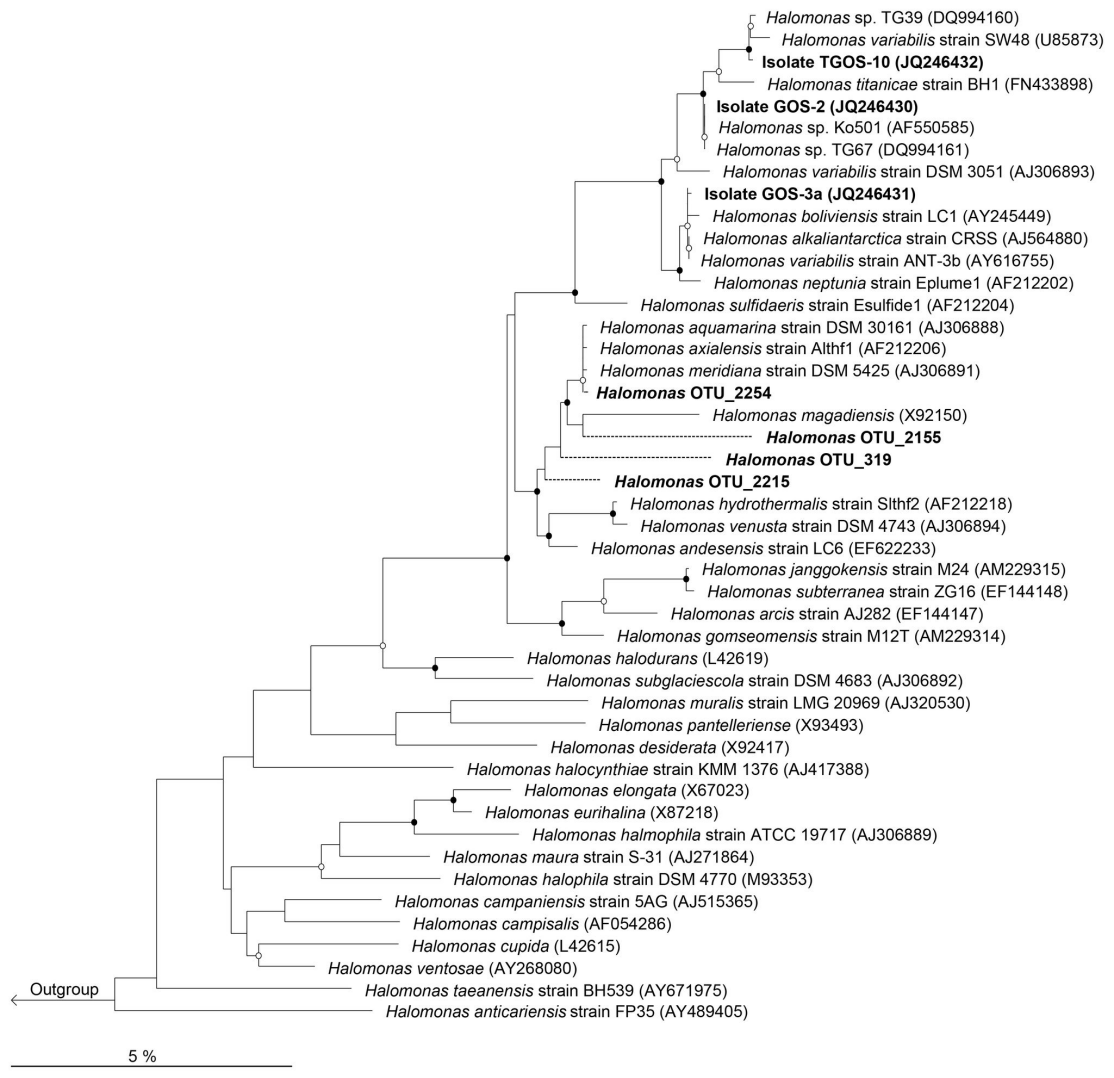
Neighbor-joining phylogenetic tree. The tree, which uses the Jukes-Cantor model of evolution, is based on 16S rRNA gene sequences (>1,200 bp) showing the relationships between isolates GOS-2, GOS-3a, TGOS-10 and representatives of related taxa. These isolates clustered together with several other EPS-producing halomonads that are marked with an asterisk. Filled circles indicate nodes with bootstrap values (1,000 bootstrap replications) greater than 90%; open circles indicate bootstrap values greater than 60%. Pyrosequence phylotypes with >97% sequence identity to any one or more of the *Halomonas* isolates were added using the ARB maximum parsimony quick-add feature (dashed branches). GenBank accession numbers are shown in parentheses. *Zymobacter palmae* (D14555) was used as an outgroup. Bar, 5 substitutions per 100 nucleotide positions.

Partial 16S rRNA gene sequences were obtained for strains GOS-2 (1442 bp), GOS-3a (1449 bp) and TGOS-10 (1443 bp). Phylogenetic analysis based on 16S rRNA gene sequences indicated that these three strains belong to the genus *Halomonas* (Figure 4). From a BLAST analysis, the highest level (100%) of sequence identity for GOS-2 was to *Halomonas* sp. strain TG67 which, like strain TG39, produces amphiphilic EPS that can effectively emulsify various types of oils [37]. Strain GOS-3a was most closely related to *Halomonas* *alkaliantarctica* CRSS [62] and *Halomonas* *variabilis* ANT-3b [31] (99.8% sequence identity), both of which are known EPS-producers, the latter of which has been reported to interface with hydrocarbons [31]. The next closest cultivated relatives to GOS-3a are *Halomonas* *boliviensis* LC1 [63] and *Halomonas* *neptuniae* Eplume1 [64] (99.7% sequence identity). Strain TGOS-10 shared 100% sequence identity to *Halomonas* sp. strain TG39, and its next closest characterized relative was the EPS-producing organism *Halomonas* *titanicae* BH1 [65] with 99.1% sequence identity.

#### Relative abundance during the spill

**Figure 5:**
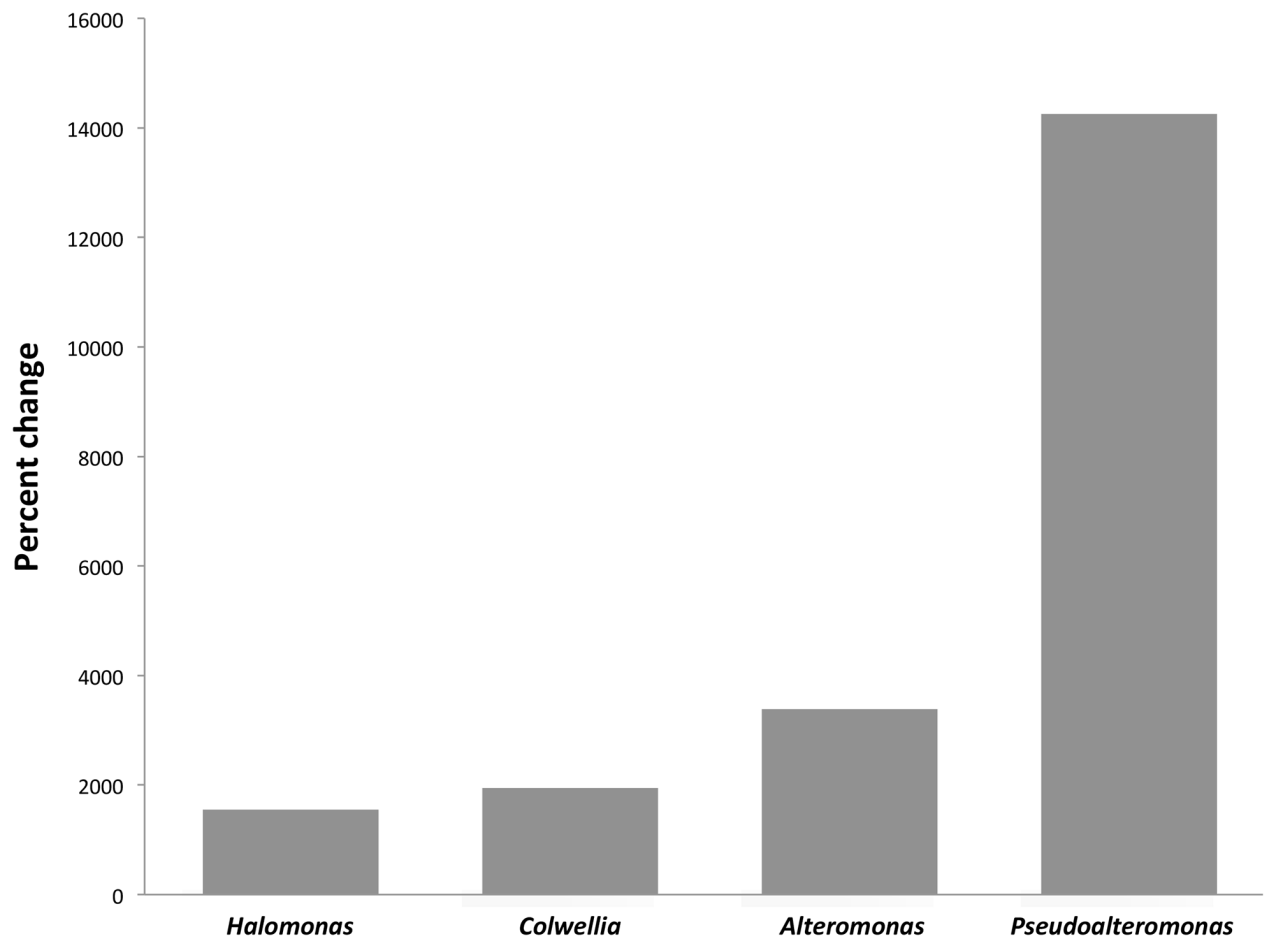
Enrichment of various exopolysaccharide (EPS)-producing bacteria during the Gulf of Mexico oil spill. Percent change in abundance of 16S rRNA gene sequences in contaminated surface water collected during the Deepwater Horizon oil spill. Changes were calculated relative to the post-spill reference sample GIP22.

Bar-coded 16S rRNA gene pyrosequencing was used to analyze the bacterial communities present in the 5 water samples from the Gulf of Mexico collected during (PE5, B1, B6, B11) and after (GIP22) the spill. A complete presentation of the community structure represented by these libraries is reported elsewhere [66], [T. Yang, L. M. Nigro, T. Gutierrez, L. D’Ambrosio, S. M. Joye, R. Highsmith, A. Teske, unpublished data]. A total of 23999, 2186, 133, 412 and 6498 high quality partial gene sequences were obtained for the PE5, B1, B6, B11 and GIP22 libraries, respectively, from which five *Halomonas* phylotypes were identified across these libraries and their relative abundances shown in Supplementary table S2. All five phylotypes were identified in the PE5 library. Of these, highest sequence similarity (≥97%) to isolate GOS-2 occurred with OTU_319 and OTU_2155; to isolate GOS-3a, with OTU_2155, OTU_2215 and OTU_2254; and to isolate TGOS-10, with OTU_319 and OTU_2215. Only one of these *Halomonas* phylotypes, OTU_2155, was identified in the GIP22 library. These and other related sequences were used to construct the neighbour-joining tree displayed in Figure 4. Our analysis did not reveal any *Halomonas* -related sequences in the B1, B6 and B11 pyrosequence libraries. The change in abundance of *Halomonas* 16S rRNA gene sequences, and of other bacterial genera recognized for their EPS-producing abilities, in contaminated surface water (PE5 sample) during the spill are shown in Figure 5, expressed relative to their abundance in the uncontaminated reference sample (GIP22). *Halomonas* , *Colwellia* , *Alteromonas* and *Pseudoalteromonas* were enriched by 1550%, 1950%, 3390 and 14250%, respectively. These organisms were not detected in the other water column samples, with the exception of *Colwellia* which was found enriched in plume water (B6 sample) by 1450% relative to the reference sample.

#### Formation of oil aggregates in roller bottles

**Figure 6:**
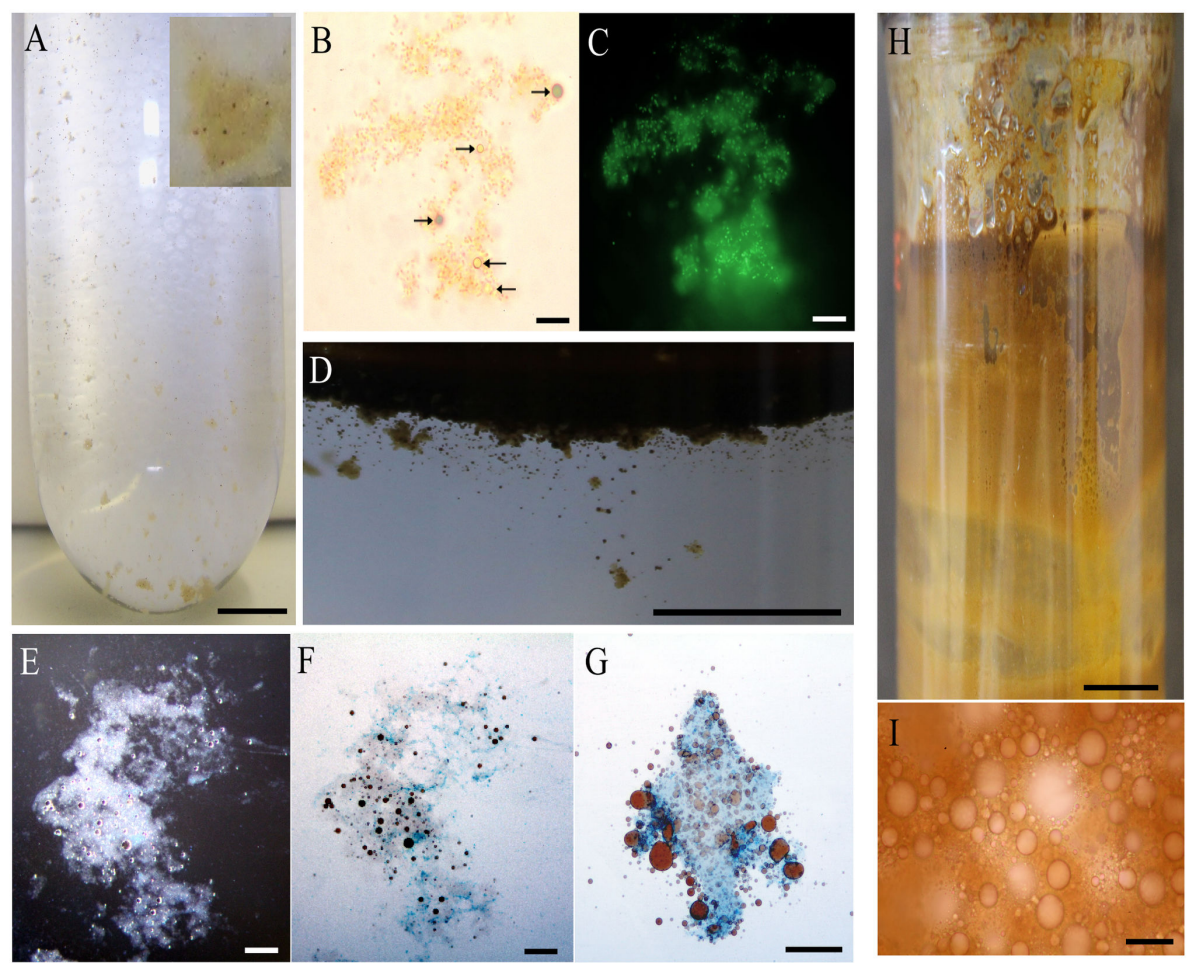
Formation of oil aggregates and/or emulsions by *Halomonas* sp. strain TGOS-10 in roller bottle incubations. Aggregates formed by inactivated (Na-azide treated; Experiment I) cells at day 3 shown sedimenting to the bottom of the roller bottle (A); *inset*, an aggregate with some associated oil droplets. An aggregate from Experiment I stained with acridine orange viewed under the light microscope (B) and then under the epifluorescence microscope with a FITC filter (C) – arrows indicate attached oil droplets. At the 12-day endpoint, all of the aggregates in this roller bottle incubation floated and some of the oil had emulsified into small droplets (D). Light microscopy revealed these aggregates contained many associated oil droplets (black-brown spheres) (E) and which stained with Alcian Blue (F) and Coomassie Brilliant Blue (G). At the 12-day endpoint, the oil in the roller bottle incubations with live cells (Experiment III) had emulsified completely and was adhered to the inner glass wall surface of the bottles (H). Under the light microscope, the emulsion was comprised of oil droplets averaging in size from <1 µm to 5 µm in diameter (I). Scale bars are 10 mm in A, D, H; 10 µm in B, C, E, F, G, I.

Roller bottle incubations using FSW amended with filter-sterilized Gulf of Mexico BP crude oil and with inactivated cells of *Halomonas* TGOS-10 (Experiment I) showed a rapid formation of oil aggregates (size range 2-4 mm) within 24 hours and their abundance increased over the proceeding 2-3 days (Figure 6A). The aggregates displayed a ‘fluffy’ off-white appearance, some with associated oil droplets, and sedimented when the bottles were held upright. Under the light microscope, some oil droplets (arrows) were observed associated with the aggregates (Figure 6B) and staining with AO revealed that the aggregates were loaded with attached cells (Figure 6C). Over the course of this experiment, the aggregates were observed to become progressively more buoyant, and by the 12-day endpoint all of the aggregates floated and settled underneath the partially emulsified oil at the top of the liquid surface when the bottles were held upright (Figure 6D). When viewed under the light microscope with the aid of dark field illumination, the aggregates appeared as an amorphous ‘cotton wool-like’ material with many associated oil droplets (dark brown spheres; average size range <1 to 5 µm i.d.) (Figure 6E) and which partially stained with AB (Figure 6F) and CBBG (Figure 6G). Conversely, no oil aggregates and emulsions formed in roller bottles incubated with cell-free fractions (Experiment II) or in control incubations with FSW+oil only or inoculated with the strain in the absence of oil (results not shown).

In roller bottle incubations with washed live cells of strain TGOS-10 (Experiment III), small particulates (≤1 mm) formed within 24 hours, but which were no longer visible thereafter (not shown). By day 3, small oil droplets formed which progressively became more abundant as the oil took on a light-brown ‘chocolate mousse’ appearance. By the 12-day endpoint, the original black oil slick in these bottles had become completely emulsified into a light brown water-in-oil emulsion that adhered tightly to the inner glass walls of the bottles (Figure 6H). Examination of the emulsion under the light microscope revealed that it was composed of water-in-oil droplets of diameters ranging from <1 to 10 µm (Figure 6I). Acridine orange stains of the emulsions and surrounding aqueous liquid revealed that cells of TGOS-10 were dispersed in the aqueous medium and not physically associated with the water-in-oil emulsion (not shown).

## Discussion

Various marine gammaproteobacteria have been found to secrete biosurfactant molecules that can enhance the solubilization of hydrocarbons. This is particularly advantageous to hydrocarbon-degrading organisms because the extremely low solubility of many hydrocarbon compounds is an important factor limiting their degradation in the marine environment [67]–[69]. Here we investigated the ability of the EPS produced by *Halomonas* sp. strain TG39 to promote the dissolution and biodegradation of hydrocarbons, with particular reference to the role that EPS-producing bacteria contributed to the fate of the oil released from the blowout of the Macondo well at Deepwater Horizon. Our initial experiments focused on evaluating the versatility of strain TG39 for degrading various hydrocarbon substrates and comparing this to the hydrocarbon-degrading profiles of other *Halomonas* species which have been shown to degrade mono-aromatic [70]–[74], polycyclic aromatic [29,36,38,75] and aliphatic [30,31,34] hydrocarbons. Notably, strain TG39 mineralized phthalate, *p*-cresol and o-cresol, which, to our knowledge, are compounds that have not previously been shown to be utilized by other species of *Halomonas* . For example, Garcia et al. [72] isolated ca. 40 distinct strains of aromatic-degrading halomonads from aquatic saltern environments and hypersaline soils in the south of Spain, none of which were found capable of degrading *p*-cresol.

Some species of *Halomonas* have been found to express EPS on their cell surface [31,34,76], which in some cases has been shown to enable the cells to make direct contact with oil droplets [31], possibly as a mechanism to access the substrate as a food source. For example, *Halomonas* sp. ANT-3b produces an emulsifying glycolipid that allows the cells to colonize and disrupt the oil–water interface of *n*-hexadecane droplets [31]. Microscopic examination of strain TG39 cells growing on *n*-hexadecane showed no evidence of their attachment to oil droplets, even after cell suspensions had been subjected to vigorous shaking under our emulsification assay (results not shown). This suggests that the cell surface of strain TG39 lacks the amphiphilic quality that is characteristic of its produced EPS. This corroborates earlier observations that showed washed whole-cell suspensions of strain TG39 to completely lack the capacity to emulsify oils, and that emulsifying activities were solely associated with cell-free extracts which contained EPS produced by the strain [37]. The *Halomonas* strains GOS-2, GOS-3a and TGOS-10 isolated from Deepwater Horizon exhibited similar characteristics, including the capacity to produce EPS that effected the emulsification of oil.

Previous work with the EPS of strain TG39 showed that it effectively emulsified *n*-hexadecane and several food-grade oils [37]. In preliminary experiments, emulsification assays performed with several other hydrocarbons, such as cycloparaffins, and mono- and alkyl-aromatics, showed this EPS to exhibit exceptional versatility in its capacity to emulsify a wide spectrum of hydrocarbon substrates (results not shown). Chemical characterization of strain TG39’s EPS revealed that its ability to associate with and emulsify hydrocarbons may be conferred by its high uronic acids (30.8%) and rhamnose content (31.7%) [37] – moieties that can render EPS quite lipophilic and mediate the adsorption of polysaccharides to oil droplets [77]–[80]. Additionally, the proteinaceous component of this EPS could also mediate its adsorption to oil droplets by nature of its hydrophobicity, penetrating into the oil where they become solvated and act as anchoring points. The observed emulsification activity prompted us to evaluate whether this polymer might enhance the solubilisation of hydrocarbons and, if so, whether this could also influence their availability for microbial degradation. The model aromatic pollutants phenanthrene, pyrene, fluorene and biphenyl were selected since these compounds have extremely low aqueous solubilities [81], making them ideal substrates for this investigation. The aromatic hydrocarbons tested covered a range of partition coefficient (log*P*) values -4.0 for biphenyl; 4.2 for fluorene; 4.5 for phenanthrene; 5.2 for pyrene [82,83]. Irrespective of ionic-strength, the effect of EPS on the aqueous solubility of the hydrocarbons correlated inversely with the inherent log*P* of these compounds. In other words, hydrocarbons with lower log*P* values were more effectively solubilised by the EPS, possibly due to physico-chemical interactions between the hydrocarbons and hydrophobic regions of the amphiphilic polymers [44,84]. The EPS-mediated enhancement in the solubilisation of hydrocarbons with lower log*P* values may be related to the polarity of these compounds, potentially allowing them to associate, to some degree, with negatively-charged residues (e.g. uronic acids) associated with the EPS [11,77]. It is noteworthy that lower soluble concentrations were measured for each hydrocarbon in the 0.6 M FSW treatment (ca. 35% salinity) compared to that in the 0.1 M treatment (ca. 0.8% salinity) at each EPS concentration used, possibly because an inverse relationship exists between salinity and hydrocarbon solubility [85]. As hydrocarbons are less soluble in seawater, the production of amphiphilic EPS by hydrocarbon-degrading bacteria in marine environments would be considered advantageous to increasing the solubility of these substrates, and thus also their bioavailability for degradation.

Interestingly, the effect of increasing concentrations of the EPS on the dissolution of biphenyl in the 0.1 M (low ionic strength) treatment did not follow the linear trend observed with the other hydrocarbons. This EPS has been shown to have a polydispersity index of 1.8, which is relatively high and denotes considerable heterogeneity in the molecular-size range of polysaccharide molecules [11]. It has also been shown that under conditions of low ionic strength, this heterogeneity is substantially reduced as polysaccharide molecules of different sizes come together to form larger molecular-weight species, or aggregates [11]. This would be expected to significantly reduce the multivalency and exposure of hydrophobic regions of the EPS that would otherwise be available to interface with hydrocarbons. The formation of these aggregates apparently occurred at EPS concentrations above 0.25 mg/ml, which would have reduced the number of biphenyl-binding sites on the EPS macromolecular structure and thereby explain the incapacity of the EPS to solubilize higher concentrations of the biphenyl. Since the rate of dissolution of a compound is a critical measure of its bioavailability [86], it can have a significant influence on the degradation rate of the compound. This is particularly important with hydrocarbons that exhibit extremely low aqueous solubilities [87,88]. Since we did not observe the attachment of strain TG39 cells to hydrocarbon droplets (results not shown), this assumes that its growth on hydrocarbons would occur at the expense of dissolved substrate [89]. Whereas the strain was unable to utilize biphenyl, fluorene and phenanthrene as sole sources of carbon and energy, its produced EPS however was found to effectively increase the solubility of these hydrocarbons. In these instances, organisms such as strain TG39 may be expected to provide an advantage to other marine bacteria that would otherwise be limited in their capacity to gain access to these compounds, as was demonstrated in our phenanthrene experiments with a natural community of indigenous microbiota from the Deepwater Horizon site. These results provide the first line of evidence that implicate *Halomonas* EPS in enhancing the degradation of oil hydrocarbons in the marine environment by increasing the dissolution and bioavailability of these compounds to indigenous oil-degrading microorganisms, such as during the Deepwater Horizon oil spill.

Though the relative abundance of *Halomonas* in oil-contaminated surface waters at Deepwater Horizon was quite low, they were by no means inactive since several *Halomonas* phylotypes were enriched (by 1550% collectively) as a result of the spill compared to the reference sample (GIP22) where all but one of the *Halomonas* phylotypes were undetected. Hazen et al. [15], for instance, also reported the enrichment of *Halomonas* by 140% in the deepwater oil plume compared to the abundance of this group in uncontaminated reference waters. The absence of 16S rRNA fragment reads in the other water column samples collected during the spill (B1, B6, B11) may be explained by the lower sequencing depth (total number of reads) in these pyrosequence libraries compared to that of the surface water PE5 library. In addition to *Halomonas* , other types of EPS-producing bacteria were markedly enriched in surface waters during the spill, which by nature of their potential to produce and release EPS, may similarly have contributed to the fate of the oil. Notably, *Colwellia* , *Alteromonas* and *Pseudoalteromonas* were enriched by at least one order of magnitude compared to their abundance in uncontaminated reference waters. These organisms collectively represented ca. 3% of total bacterial sequences in the PE5 library. Since strains of these genera have been well characterized for their ability to produce EPS [6,90]–[95], their enrichment in surface waters during the spill may have also stimulated their production and release of significant quantities of EPS. Considering the potential of marine bacterial EPS to interface with oil hydrocarbons and enhance the bioavailability of these compounds for microbial degradation, we posit that these types of bacteria contributed significantly to the fate of the oil released into the Gulf of Mexico via their production and release of EPS.

Supporting this hypothesis, insights on the formation of oil aggregates during the Gulf spill also point to a role for bacterial EPS in the fate of the oil. Oil-in-water emulsions at the sea surface-air interface were observed during the Gulf spill [24] and were very likely a precursor to the formation of oil aggregates that were found prolific in surface and, to a lesser extent, in plume waters [25,28]. Considering that oil aggregates were observed before BP had begun dumping tonnes of dispersant into the Gulf of Mexico [28], and laboratory roller-bottle experiments showed their rapid formation in the absence of any added dispersant [25], the formation of these aggregates was very likely a biologically-driven process, possibly initiated via the physicochemical interaction between oil droplets, microorganisms and biopolymer(s), likely of bacterial origin [15,28]. Ziervogel et al. [25] showed that oil aggregates yielded high levels of peptidase and β-glucosidase activities, indicating that a major component of their composition is glycoprotein. This is consistent with the composition of marine bacterial EPS [6,96,97], including that from many *Halomonas* species such as strains TG39 and TG67 [37] – strains with 100% 16S rRNA gene sequence identity to, respectively, the strains TGOS-10 and GOS-2 which we isolated from the Gulf spill site. During incubations with uncontaminated deep water samples collected during the active phase of the Gulf oil spill, Baelum et al. [98] reported on the formation of flocs (synonymous with oil aggregates). Analysis of these flocs revealed that they were composed of oil, carbohydrates and biomass, predominantly of bacteria most closely related to *Colwellia* . Members of *Colwellia* have been shown to produce EPS under conditions reminiscent of those found in cold deep Gulf of Mexico waters [93], suggesting that *Colwellia* may have contributed to the fate of the oil, including formation of oil aggregates, through their production of EPS during the Gulf of Mexico spill. Since these experiments were conducted using mixed microbial communities, it is difficult however to ascertain the formation of these flocs to *Colwellia* species. No methods currently exist that can accurately trace the production of polymeric substances to their biological source in environmental samples, which therefore circumvents our ability to explain what type(s) of polymeric substances participated in the formation of flocs or oil aggregates, and to consequently link them to a producing organism(s).

Using a roller bottle incubation design [25], we demonstrated the independent ability of *Halomonas* strain TGOS-10 to promote the formation of oil aggregates and emulsification of the BP crude oil. We showed that the extracellular cell surface of this organism played a direct role in aggregate formation, as demonstrated in roller bottle incubations using non-respiring cells that had been rendered inactive by treatment with sodium-azide. Baelum et al. [98] proposed that aggregate formation was initiated on the surface of oil droplets. However, direct microscopic observation of nascent aggregates that formed in the first 3 days of our roller bottle incubations contradicts this because some of these aggregates lacked associated oil droplets. Oil droplets, therefore, likely did not act as a nucleation point for aggregate formation. A distinctive feature of the aggregates was their heavy loading with attached TGOS-10 cells, indicating that bacterial cell-surface-expressed EPS potentially contributed a key role in aggregate formation. Hence, the combination of bacterial cells (i.e. strain TGOS-10), dissolved hydrocarbons and EPS were likely involved in having promoted the formation of mucilaginous aggregates that progressively adopted associated oil droplets – the latter conferring the aggregates with increased buoyancy and eventual ability to float. Positive staining of the aggregates with AB and CBBG provided evidence of their glycoprotein composition, which is a major component of marine bacterial EPS [6,96,97].

The ephemeral formation of inconspicuous aggregates in the first 24 hours of the roller bottle incubations with respiring cells was thereafter overshadowed by the emulsification of the oil slick, progressively becoming a reddish-brown that is reminiscent of weathered oil after an oil spill at sea [99]. Preliminary experiments with strain TGOS-10 showed it capable of degrading *n*-alkanes and aromatic hydrocarbons (results not shown), suggesting that the extensive emulsification of the oil in these roller bottle incubations could be attributed to the breakdown of biodegradable components in the oil, such as low molecular weight aliphatic and aromatic hydrocarbons. The more recalcitrant components of oil (i.e. asphaltenes and resins) are often the remnants of weathered oil. By nature of their polarity, they can form stable water-in-oil emulsions similar to that observed in these roller bottle incubations. Since the formation of aggregates preceded the emulsification of oil in the roller bottle incubations that contained inactivated (Experiment I) or active (Experiment III) cells, and aggregates did not form in control incubations without bacterial cells added, this indicates that cell-bound EPS of strain TGOS-10 contributed to promoting aggregate formation.

In conclusion, we showed that the EPS produced by *Halomonas* sp. strain TG39 can effectively mediate the dissolution of poorly-soluble aromatic hydrocarbons and enhance their bioavailability to indigenous populations of microorganism that were present during the active phase of the oil spill in the Gulf of Mexico. As this model strain shared 100% 16S rRNA gene sequence identity to TGOS-10 and their produced EPS exhibited similar physicochemical properties, these and other EPS-producing halomonads enriched during the spill likely contributed to the degradation of the oil and in oil-aggregate formation. Moreover, species of *Colwellia* , *Alteromonas* and *Pseudoalteromonas* , which were also found enriched in surface and deep waters during the spill, may also have contributed to the formation of oil aggregates by nature of their potential to produce EPS. There is substantial evidence to suggest that oil-aggregate formation during the Gulf spill was mediated by bacterial EPS, either produced in response to the oil released, or that was present as part of the pre-spill DOM pool in the water column. Importantly, our results provide evidence that this bacterial-derived EPS contributed to the dissolution, bioavailability and microbial degradation of aromatic hydrocarbons, particularly in surface waters where EPS-producing bacteria were preferentially enriched. While whole bacterial and eukaryotic phytoplankton cells may also play a role [28], these results add to a growing body of evidence to implicate bacterial-derived EPS in the fate of the oil released at the Deepwater Horizon site.

## Supporting Information

Table S1 Rates and extent to which phenanthrene was degraded during incubation of the Gulf oil spill microbial community on increasing concentrations of exopolysaccharides (EPS) from *Halomonas* sp. strain TG39. (DOCX)

Table S2 Relative abundance (%) of *Halomonas* phylotypes identified in 16S rRNA gene pyrosequence libraries. (DOCX)
