# Supplementary material for: Role of Bacterial Exopolysaccharides (EPS) in the Fate of the Oil Released during the Deepwater Horizon Oil Spill
Source: PLoS One. 2013 Jun 27;8(6):e67717. doi: 10.1371/journal.pone.0067717 (PMC3694863; doi:10.1371/journal.pone.0067717)
Supplement: Table S1 — (DOCX) [file pone.0067717.s001.docx]

**Supplementary table S1.** Rates and extent to which phenanthrene was degraded during incubation of the Gulf oil spill microbial community on increasing concentrations of EPS from *Halomonas* sp. strain TG39.

Incubations using Gulf oil-spill water

EPS Lag Rate at Rate at Extent degraded

(mg/ml) (days) 2-5 days 5-8 days after 12 days

(mg/l/day)^a^ (mg/l/day)^a^ (%)^b^

0.0 2 210.6 ± 18.1 475.9 ± 30.7 100.0 ± 0.0

0.1 2 288.6 ± 22.9 528.1 ± 37.7 100.0 ± 0.0

0.2 2 281.0 ± 75.9 536.9 ± 74.6 100.0 ± 0.0

0.4 2 309.9 ± 39.3 526.2 ± 56.6 100.0 ± 0.0

^a^ Values are the slopes of a linear fit ± 95% confidence interval.

^b^ Percentage degraded of total initial phenanthrene.
