# Supplementary material for: Role of Bacterial Exopolysaccharides (EPS) in the Fate of the Oil Released during the Deepwater Horizon Oil Spill
Source: PLoS One. 2013 Jun 27;8(6):e67717. doi: 10.1371/journal.pone.0067717 (PMC3694863; doi:10.1371/journal.pone.0067717)
Supplement: Table S2 — (DOCX) [file pone.0067717.s002.docx]

**Supplementary table S2.** Relative abundance (%) of *Halomonas* phylotypes identified in 16S rRNA gene pyrosequence libraries.^a^

During spill (May 2010): After spill (Oct. 2010): Isolates with >97%

OTU_ID PE5 B1 B6 B11 GIP22 (Control) 16S rRNA similarity

319 0.03 0 0 0 0 GOS-2, TGOS-10

801 0.02 0 0 0 0 none^b^

2155 0.01 0 0 0 0.01 GOS-2, GOS-3a

2215 0.03 0 0 0 0 GOS-3a, TGOS-10

2254 0.05 0 0 0 0 GOS-3a

**Total (%): 0.14 0.0 0.0 0.0 0.03**

^a^ Percentage of *Halomonas* sequences of total reads in each library.

^b^ Low 16S rRNA sequence identity (<95%) to *Halomonas* isolates GOS-2, GOS-3a and TGOS-10.
